# Binary and Ternary Blended Portland Cements Containing Different Types of Rice Husk Ash

**DOI:** 10.3390/ma17122923

**Published:** 2024-06-14

**Authors:** Luis Miguel Ordoñez, María Victoria Borrachero, José Monzó, Lourdes Soriano, Jordi Payá

**Affiliations:** 1 KhemeChemical S.L., Puerto de Sagunto, 46520 Valencia, Spain; lmordonez@khemechemical.com; 2 Institute of Science and Technology of Concrete (ICITECH), Universitat Politècnica de València, 46022 Valencia, Spain; vborrachero@cst.upv.es (M.V.B.); jmmonzo@cst.upv.es (J.M.); lousomar@upvnet.upv.es (L.S.)

**Keywords:** rice husk ash, pozzolanic activity, workability, compressive strength, ternary cement

## Abstract

Rice husk ash (RHA) is agricultural waste with high silica content that has exhibited proven technical feasibility as a pozzolanic material since the 1970s. Notwithstanding, its use in mortars and concrete is limited by the standards currently utilized in some countries where RHA production is high and the aforementioned pozzolanic material is not standardized. This is the case in Spain, one of the main rice producers in Europe. Nowadays, the high pressure placed on the Portland cement production sector to reduce its energy use and CO_2_ emissions has given rise to a keen interest in mineral admixtures for cement manufacturing. In this research, we intended to establish the contributions of different RHA types to the final blended Portland cement properties (“H” is used to identify RHA in standardized cements). The experimental results demonstrated that RHA with good pozzolanic properties (large specific surface and high amorphous silica content) had to be limited to 10% cement replacement because of the severe reduction in workability at higher replacement percentages. RHA with lower reactivity, such as crystalline RHA, or fly ash (FA) can be used to prepare binary and ternary blended cements with reactive RHA. It is possible to design the following cements: CEM II/A-H and CEM II/A-(H-V). It would also be possible to design cement (CEM II/B-(H-V) with replacement values of up to 30% and the same 28-day mechanical performance as observed for the Portland cement without mineral addition.

## 1. Introduction

The construction industry is a large consumer of non-renewable raw materials. In addition, cement manufacturing produces 5–7% of the global industrially emitted CO_2_ [1]. The cement production in 2022 amounted to 4160 Mt, and this production is expected to reach 4260 Mt in 2030. The clinker-to-cement ratio (ton per ton) in 2022 was 0.71, and it is likely to lower to a value of 0.57 by 2050 [2]. De Brito and Kurda [3] point out several solutions to reduce the total binder amount: (i) the use of supplementary cementing (pozzolanic or hydraulic) materials; (ii) the use of fillers; (iii) the impact on the water/cement ratio and the use of chemical additives; and (iv) an indirect reduction in the binder amount.

Regarding the use of supplementary cementing materials—specifically pozzolanic materials—one of the most exciting groups is that composed of biomass-burning ashes [4]. Agricultural waste is a sustainable energy resource, and some ash types that are derived from biomass burning have pozzolanic characteristics [5,6]. Some, like rice husk ash (RHA), have been studied for decades [7,8,9].

Rice husk is an agro-waste material that is produced worldwide in large amounts. According to the literature, the global forecast for 2021/2022 was 714.2 million tons, which is equivalent to around 142.84 million tons (ca. 20% of paddy rice) of rice husk [10]. Most of this waste is produced in undeveloped Asian countries (India, China, Indonesia, Bangladesh and Thailand). In Europe, Spain is one of the most important rice producers, and 381.3 thousand tons of paddy rice grain were produced in 2022 [11]. This means that around 80,000 tons of rice husk is available in Spain for valorization. Rice husk contains [12,13] organic substances (cellulose, lignin, fiber) and 20% of inorganic material, mainly silicon dioxide (SiO_2_ > 85%). There are two means of removing organic matter from rice husk: (a) combustion with energy recovery and (b) simple incineration. Endale et al. [10] report that, for 0.2 tons of rice husk, around 0.05 tons (ca. 20% of the yield) of RHA is produced. This means that around 15,000–20,000 tons of ash would be produced in Spain. Thus, the potential RHA production worldwide would be 25–30 million tons.

RHA’s pozzolanic activity depends on several parameters, mainly those related to the reached temperature and the residence time at high temperatures. The most important properties of RHA that determine its pozzolanic activity are the amorphous phase content and specific surface area. When the combustion temperature exceeds 800 °C and the residence time at temperatures higher than 800 °C is long, partially/totally crystallized RHA is obtained, and the specific surface area diminishes. Consequently, its pozzolanic activity reduces. RHA is obtained by controlled burning methods with the monitoring of the temperature, where the resulting ash is quickly removed from the furnace. The controlled conditions make it possible to obtain RHA with excellent pozzolanic behavior. Under uncontrolled burning conditions, the temperature could reach more than 1000 °C due to the intense exothermic oxidation of the rice husk’s organic matter, and the crystallization process is easily achieved, resulting in the formation of cristobalite and tridymite [14,15,16]. Regarding the fresh properties, concrete or mortar mixtures containing RHA usually obtain lower slump and fresh and dry density values [17,18]. In the hardened state, mixtures containing RHA have higher compressive strength values and improved durability [19,20,21].

The design of ternary blended cements is a focus of interest because this combination enhances the sustainability [22], workability [23] and mechanical/durability properties [24].

Depending on the RHA’s properties, it is possible to prepare different blended cements based on a Portland cement clinker. Furthermore, distinct strength category types of cement could be designed. The goal of this research is to assess the workability and compressive strength of mortars prepared with cements containing different types of RHA and other mineral admixtures. Based on these characterizations, a proposal for the denomination of potential standardized blended cements is given. Different RHA blending percentages are studied to prepare the optimum blended cements. The synergistic effects on the blended cements based on highly reactive RHA and other mineral admixtures, such as low-reactivity RHA, fly ash (FA) or ground granulated blast furnace slag, are evaluated. Mortars prepared with the RHA-blended cements, with good performance in terms of workability and strength, are proposed.

## 2. Materials and Methods

**Figure 1:**
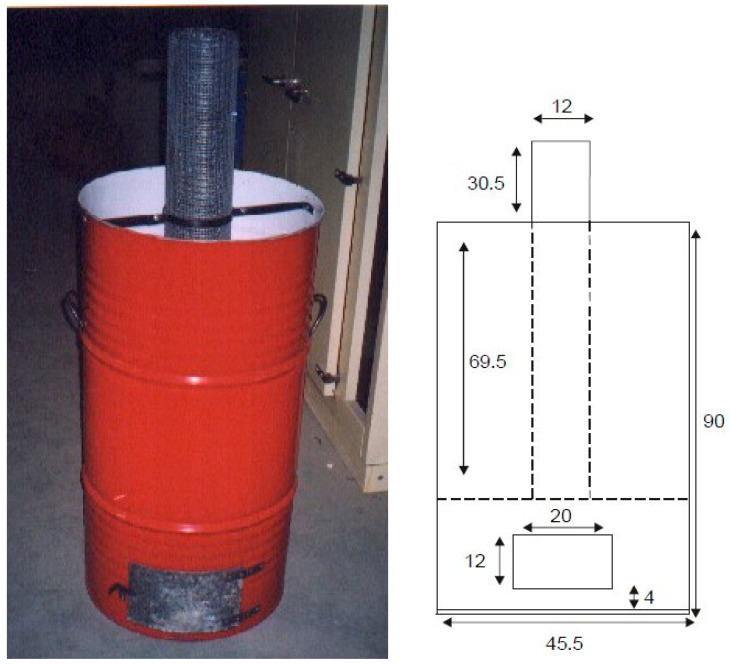
Small incinerator prototype (on the right; all data are expressed as mm).

The ordinary Portland cement (OPC) used in the experiment was the UNE-EN 197-1 CEM I-52.5-R type [25]. It was supplied by Cemex (Buñol, Spain), while the crystalline (RHA-1 and RHA-2) and semicrystalline (RHA-3) RHA samples were supplied by Agrocítrica (Alzira, Spain) and NK Enterprises (Jharsuguda, Odisha, India), respectively. The amorphous RHA sample (RHA-4) was obtained under controlled combustion conditions using a small incinerator prototype (Figure 1). FA (F type, Andorra Thermoelectric Power Plant, Andorra-Teruel, Spain), non-densified silica fume (NDSF; Ferroatlántica S.A., A Coruña, Spain), ground granulated blast furnace slags (GGBFS-1 and GGBFS-2; Dortmund, Germany) and silica flour QF (quartz S100 type, Sibelco, Arcos de la Frontera, Spain) were also used. The fine aggregate was standard siliceous sand according to the CEN standards (>99% quartz) [26].

The RHA samples were ground using a laboratory ball-mill (Gabbrielli, Calenzano, Italy) with 18-mm-diameter alumina balls. The blended cements were prepared by previously mixing ground additions (if necessary) and OPC, following the recommendations of Cook [7].

The total silica content in the mineral admixtures was determined by X-ray fluorescence using the Philips MagiXPRO equipment (Philips Analytical, Almelo, The Netherlands). Lithium tetraborate was employed to prepare the samples and the solid mixture was melted in the Vulcan 4M—Fusion Machine Type VAA 4 equipment (Fluxana GmbH & Co., Bedburg-Hau, Germany). The amorphous silica in the mineral admixtures was determined by the method described by Payá et al. [27]. The BET specific surface area (nitrogen adsorption) was measured by a MicromeriticsTriStar 3000 model (Micromeritics, Norcross, GA, USA).

The compressive strength was determined in prismatic test specimens (40 × 40 × 160 mm) according to [26]. These specimens were cast from a batch of plastic mortar containing cement/water/sand in proportions of 1.0:0.5:3.0 by mass. The mortar was prepared by mechanical mixing. Before molding, the cement’s workability was determined according to UNE-EN 413-2:2017 [28]. The specimens in the mold were stored in a moist atmosphere for 24 h at 20 °C. Then, the demolded specimens were stored under lime-saturated water at 20 °C until strength testing. At the required age, the specimens were taken from their wet storage and broken flexurally into two halves, and each half was tested for its strength in compression.

## 3. Results and Discussion

### 3.1. Physico-Chemical Properties of RHA Samples

**Figure 2:**
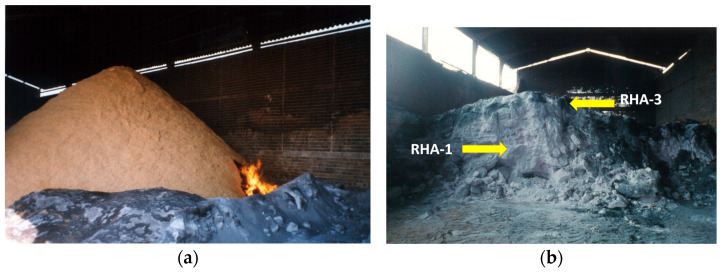
Open-field combustion in a very well-ventilated shed for samples RHA-1 and RHA-2: (**a**) at the beginning of burning; (**b**) after burning had ended and part of the ash had been removed. Arrows indicate the zones where RHA-1 and RHA-2 were sampled.

Table 1 summarizes the main physico-chemical properties, which defined the reactivity of the RHA samples and the other materials used therein. Four RHA samples (the chemical compositions of these samples are given in Appendix A—Table A1; the XRD diffractograms are depicted in Appendix B—Figure A1) were selected for the experimental studies: two (RHA-1 and RHA-2) presented high crystallinity because the amorphous silica percentage in relation to the total silica was less than 25%. This characteristic was related to the ash-yielding method: an open-field incineration process without energy recovery (Figure 2). Under this condition, the temperature reached by the ash was above 900 °C and remained high for several hours. A high combustion temperature and residence time activate amorphous silica crystallization to produce cristobalite and tridymite [7,29]. An RHA sample produced at a controlled combustion temperature (RHA-3) was supplied by NK Enterprises (India). This sample presented 71% amorphous silica in relation to the total silica. Finally, a fourth RHA sample was obtained by controlled combustion in a prototype incinerator (Figure 1). This sample presented 100% amorphous silica in relation to the total silica because the combustion temperatures did not reach 700 °C. RHA’s pozzolanic efficiency depends on the amorphous SiO_2_ content. Uncontrolled incineration processes provide high temperatures and long residence times for incineration (open-field combustion). The ashes obtained under these conditions are crystalline (formation of tridymite and cristobalite [29]). These samples obtained low loss on ignition (LOI) values because the carbon content was removed as CO_2_. RHA-1 and RHA-2 had LOI values below 5% (0.14% and 4.52%, respectively), which demonstrated the effect of the high temperature and long residence time. The LOI values for RHA-3 and RHA-4 were significantly high (8.24% and 17.65%, respectively), which agreed with the low temperature reached during the combustion process. In this case, part of the husk’s organic matter was converted into carbon particles [30].

RHA’s reactivity also depends on the specific surface area (measured by the BET method), and this parameter is also related to the combustion conditions. Hence, the samples obtained at high temperatures and long residence times had low BET values: 1.6 m^2^/g for RHA-1 and 9.3 m^2^/g for RHA-2. RHA-1 was collected from the central zone of the ash pile, whereas RHA-2 was collected from the upper zone, which was the reason for the difference in the BET values. The RHA samples obtained under controlled conditions obtained significantly higher BET values: 15.2 m^2^/g for RHA-3 and 36.5 m^2^/g for RHA-4.

**Figure 3:**
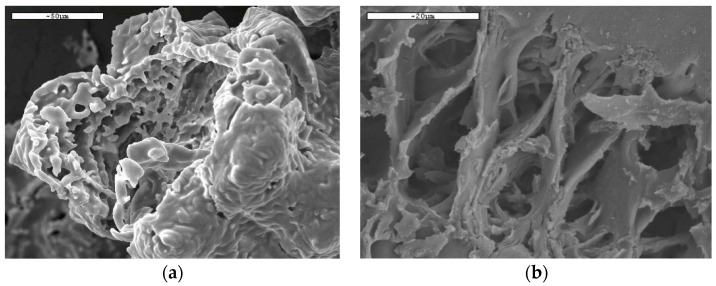
SEM micrographs for (**a**) RHA-1, scale bar 50 µm; (**b**) RHA-4, scale bar 20 µm.

**Figure A2:**
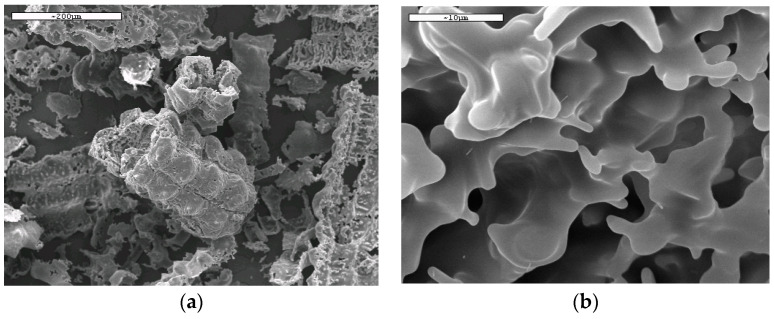
SEM micrographs for RHA-1: (**a**) ×200; (**b**) ×3500.

**Figure A3:**
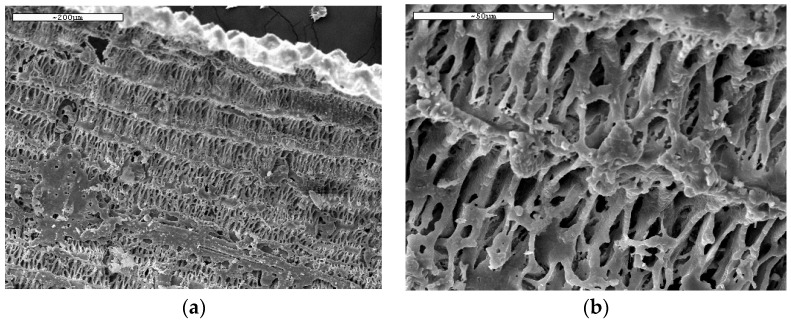
SEM micrographs for RHA-2: (**a**) ×200; (**b**) ×2000.

**Figure A4:**
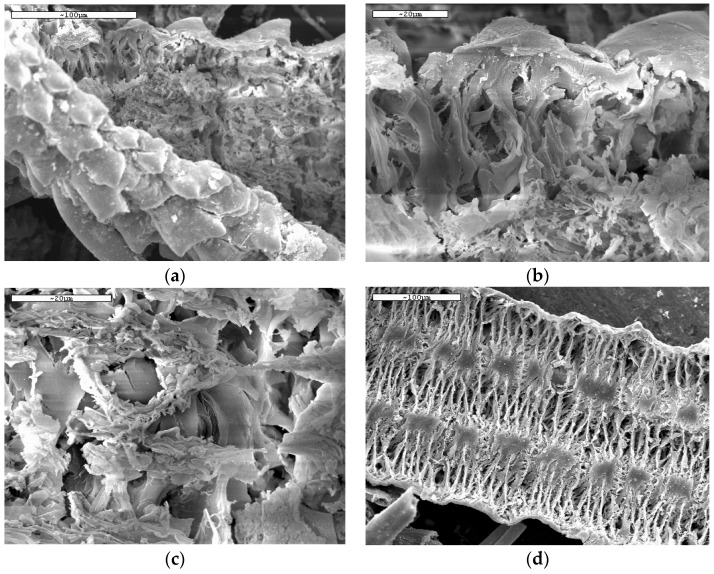
SEM micrographs for RHA-4: (**a**) ×500; (**b**) ×1500; (**c**) ×2000; (**d**) ×350.

RHA-1 and RHA-4 were compared using SEM. The micrographs (Figure 3a,b) showed differences in the internal structures of the non-ground ash particles. The particle in Figure 3a presents a round-shaped internal structure, which is indicative of silica coalescence [31] due to high temperatures, unlike Figure 3b, which shows an unaltered internal silica skeleton of husk without coalescence. Additional micrographs are presented in Appendix C (Figure A2, Figure A3 and Figure A4).

As shown in Figure 3, the RHA particles had a high internal porosity, which is not ideal for blending with PC. This is because it leads to the loss of workability of fresh mixes. For this reason, the RHA samples were ground to achieve a similar particle distribution to OPC. Table 1 summarizes the granulometric parameters: the percentage within the 3–32 µm range fell within the 62–73% interval for RHAs and was slightly lower than that for the PC type CEM I 52.5 R (83.8%).

Table 1 also summarizes some physico-chemical parameters for FA, NDSF, GGBFS-1 and GGBFS-2 and QF. There are similarities in the amorphous silica content and the specific surface between RHA-4 and NDSF and also between RHA-1 and QF.

### 3.2. Influence of RHA on Compressive Strength Development

At this stage, samples RHA-1, RHA-3 and RHA-4 were selected to prepare blended cements by mixing CEM I 52.5R with the RHAs in the RHA/CEM I proportions of 5/95, 10/90, 15/85 and 20/80. The standardized mortars [26] were prepared and tested under compression after 2, 7 and 28 curing days.

**Figure 4:**
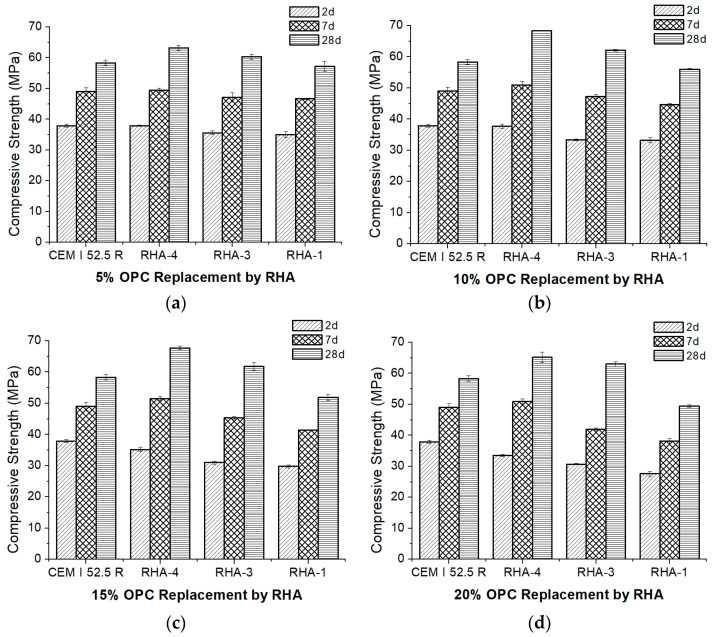
Compressive strength development for the control mortar (CEM I 52.5R) and the RHA mortars with different replacement levels: (**a**) 5%; (**b**) 10%; (**c**) 15%; (**d**) 20%.

Figure 4 shows the compressive strength values obtained for the cured mortars according to the level of replacement of cement with RHA. The mortars containing RHA-4 displayed the best behavior compared to the other RHA samples. Thus, for the 5% and 10% replacement levels, the compressive strengths obtained for all curing ages (2, 7 and 28 days) equaled and/or exceeded the values for the CEM I 52.5R mortar (control), which demonstrated the high pozzolanic reactivity of RHA-4. For the highest replacement levels (15% and 20%), the contribution of RHA-4 for curing ages of 7 and 28 days was very important and they equaled or surpassed the control mortar’s strength. At 2 curing days, the compressive strengths for the 15% and 20% replacements were lower than for the control. This was attributed to both the reduction in clinker content and the limited pozzolanic reaction development at an early age due to the low portlandite production from the hydration of calcium silicates in the clinker.

The contribution of the strength development of the mortars with RHA-3 and RHA-1 was significantly less, and the control specimen’s compressive strength was achieved only for the mortars containing RHA-3 for the 28-day curing period (for all replacement percentages).

The different pozzolanic reactivity of the RHA samples (RHA-4 > RHA-3 > RHA-1) was clearly evidenced by the compressive strength values obtained after 28 curing days and for all replacement percentages. An optimum compressive strength value at 28 curing days when 10% OPC was replaced with RHA-4 was observed. The pozzolanic reaction rate depends on the available portlandite released during OPC hydration. When 20% OPC was replaced with RHA-4, the quantity of released portlandite decreased by approximately 20%. This pozzolan reacts with portlandite at early ages due to its high reactivity. Both effects caused the pozzolanic reaction efficiency to decrease more quickly because the CEM I 52-5R replacement level was higher [15,32].

Ganesan et al. [32] studied the reactivity of an RHA sample with a similar specific surface area (36.47 m^2^/g) to RHA-4 and low crystallinity. These authors prepared mortars with the replacement of ordinary Portland cement in the range of 0–35%, and they found that the optimum replacement was 15% for the 28-day compressive strength. Similarly, RHA-4 mortars with 10 and 15% replacement yielded the highest values at 28 days. Distinctively, the behavior found for early ages was different: Ganesan et al. [32] found that, at 1 and 3 days of curing, the optimum was also 15% replacement. However, the compressive strength of RHA-4 mortars at 2 days of curing decreased slightly with the increase in the replacement percentage. This difference at an early curing age is attributed to the RHA fineness: the ash used by Ganesan et al. had a mean particle size of 3.80 µm (six times finer than Portland cement), whereas RHA-4 presented a similar particle distribution to CEM I 52.5 R (Table 1). When the RHA particle size is lower, the pozzolanic reactivity occurs earlier, and the contribution to strength development is more important at early curing ages.

The differences in reactivity among the studied RHAs are related to the potential pozzolanic reactivity derived from the physico-chemical characterization (see Table 1). The granulometric parameters are similar for RHA-1, RHA-3 and RHA-4, and the compressive strength values of the mortars depicted in Figure 4 are attributed to the relative amorphous silica content (100% for RHA-4; 71% for RHA-3; 11.7% for RHA-1) and the specific surface area (36.5 m^2^/g for RHA-4; 15.2 m^2^/g for RHA-3; 1.6 m^2^/g for RHA-1). Thus, higher amorphous silica content and a larger specific surface area yielded better compressive strength in the RHA mortars.

### 3.3. Cement Types according to RHA Reactivity and Standards

The classification of conventional and standardized blended cements is established by the European Standards Association [25]. It considers the following information/descriptions.

- Cement design: CEM type/proportion (types of addition) + strength category (taking into account long and short curing ages).
- Type: I (additions below 5%), II (OPC with additions up to 35%), III (OPC with GGBFS), IV (pozzolanic cements) and V (composed cements).
- Proportion of addition: the classification depends on the type of cement (II, III, IV or V). For CEM II, the CEM II/A type has 6–20% clinker replacement and the CEM II/B type has 21–35% clinker replacement.
- Types of addition: limestone filler (L), FA (V), GGBFS (S), silica fume (D), among others.
- Mechanical strength category: 32.5, 42.5 or 52.5 compressive strength values at 28 days in standardized mortar. Type N and type R, respectively, indicate low- and high-rate strength development after 2 or 7 curing days in standardized mortar (Table 2).

**Table 2:** Compressive strength categories of standardized cements according to UNE-EN 197-1 [25].

Category	Mechanical Compressive Strength MPa (N/mm^2^)			
	2 Days	7 Days	28 Days	
32.5 N	--	≥16.0	≥32.5	≤52.5
32.5 R	≥10.0	--		
42.5 N		--	≥42.5	≤62.5
42.5 R	≥20.0	--		
52.5 N		--	≥52.5	--
52.5 R	≥30.0	--		

For instance, CEM I-52.5-R was composed of 95–100% clinker and provided more than 30 and 52.5 MPa compressive strength at 2 and 28 days, respectively, in the standard mortars. Another example could be CEM II/B-(L-V) 32.5 N, composed of 21–35% FA plus limestone filler, which provided more than 16 and 32.5 MPa compressive strength at 7 and 28 days, respectively.

However, RHA use is not permitted as mineral addition during cement formulation. In the future, it is likely that RHA will be one of the candidates to be considered for mineral addition in cement because the availability of slag, and especially of FA, is being questioned for sustainability and climate change reasons [33]. If we use the symbol “H” to denote the cements with RHA, the following cements could be prepared after taking into account the RHA samples analyzed in the previous section (Table 3). For the RHA-blended cements, clinker replacement above 20% could not be considered because the resulting cement would perform poorly in terms of workability. This effect has also been observed by Kamau et al. [34]. Thus, only the CEM II/A-type cements can be considered regarding the assignment of nomenclature to the new blended cements with RHA and, consequently, the CEM II/B and CEM IV or V types cannot be contemplated.

In general, when high-reactivity RHA is used (e.g., RHA-4), CEM II/A-H 52.5 R can be prepared. However, for RHA-3, the strength category lowers to 52.5N when high replacement levels (15–20%) are selected. Finally, for the RHA with the lowest reactivity (RHA-1), CEM II/A-H 52.5 N was prepared with less than 10% RHA, and the strength category was lowered to 42.5 R for the highest replacement levels (15% and 20%).

### 3.4. Comparison of Contribution of RHA and Other Mineral Admixtures to Compressive Strength Development

**Figure 5:**
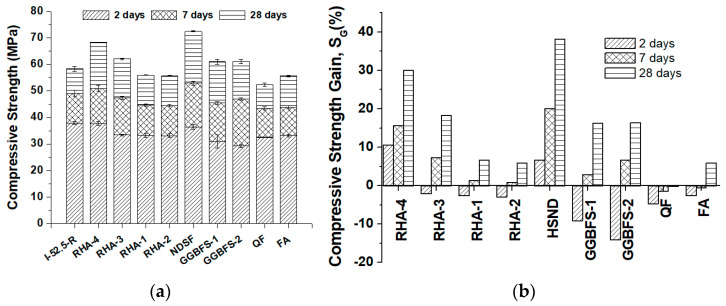
Strength development of the blended cements (10% replacement level): (**a**) compressive strength; (**b**) compressive strength gain (S_G_).

Reactivity studies of different mineral admixtures in terms of the mortar’s compressive strength performance were carried out to perform a comparison in terms of the RHA contribution. The cements were CEM I 52.5 R (control mortar) and the blended cements in which 10% of CEM I was replaced with the corresponding mineral admixture. Four RHA samples (RHA-1, RHA-2, RHA-3, RHA-4) were tested. Additional cements were prepared using GGBFS-1 (4000 cm^2^/g Blaine), GGBFS-2 (5000 m^2^/g Blaine), FA, NDSF and QF. Figure 5 depicts the compressive strength values of the blended cements (10% replacement) for 2, 7 and 28 curing days. The compressive strength gain [35] values are also shown. The strength gain (S_G_, %) was calculated as follows:

(1) $$S_{G}\;\left(\%\right)=\frac{S_{i}-\left[(P*S_{c})/100\right]}{\left[(P*S_{c})/100\right]}*100$$

where S_c_: compressive strength of the control mortar (100% CEM I 52.5 R); S_i_: compressive strength of the blended cement; P: percentage of replaced CEM I in the mortars containing the mineral admixture.

The 28-day compressive strength values obtained in the blended cements with RHA-1, RHA-2 and FA were similar at all curing ages. However, the compressive strength obtained using quartz flour (QF) was lower. This behavior suggests the minor contribution of the pozzolanic and/or physical effects to the strength gain (5–8% after 28 curing days) when using either 10% RHA-1 or 10%RHA-2.

The compressive strength and mechanical gain values at the earliest curing age (2 days) are noteworthy as they were higher when RHA-4 was used in the blended cements than when employing NDSF. The main reason for this was the larger specific surface area when utilizing RHA-4. Xu et al. [36] reported a higher strength value for a mortar with RHA at 3 days (44.23 MPa) than for a silica fume one (39.43 MPa). The opposite effect was observed at 28 curing days, when the compressive gain was 10% higher when NDSF was used. In this case, the higher LOI value for RHA-4 (17.65%) and the larger number of particles smaller than 3 microns in NDSF (aggregate–paste interface refinement) were the reasons for this behavior at longer curing times. Xu et al. [36] also reported higher strength for a silica fume mortar (75.59 MPa) than for an RHA one (71.02 MPa) at 28 curing days.

Applying GGBFS delayed the early clinker hydration stages because a negative compressive strength gain of 10% and 15% for GGBFS-1 and GGBFS-2, respectively, was noted (Figure 5b). However, between 2 and 7 curing days, the compressive strength gain was higher due to the hydraulic reactivity of these admixtures. Finally, at 28 curing days, the compressive strength for the control mortar was achieved using 10% GGBFS replacement, which yielded 15% S_G_. The observed mechanical behavior when using 10% GGBFS-2 was similar to that observed for RHA-3 from 7 to 28 curing days. The strength development of the cement containing QF showed that its contribution was negligible: the S_G_ value for 28 curing days was close to zero.

Thus, in terms of compressive strength development, for the period of 28 curing days, the RHA-4 sample was similar to NDSF, RHA-3 was similar to GGBFS, and RHA-1 and RHA-2 were similar to FA.

### 3.5. Workability and Mechanical Properties of Standardized Blended Cement Containing RHA

In practical terms, additional information may be supplied for the blending of cements, especially aspects related to the water demand of the new blended cements. It is well known that some mineral additions play a water-reducing role when mixed with OPC, e.g., FAs [37,38]. However, when mineral admixtures with a large specific surface area (e.g., silica fume) are blended, the water demand is likely to increase [39,40], to such an extent that it is not feasible to prepare mixtures with appropriate workability at high replacement percentages. In this context, workability studies have been carried out using a flow table [28] for some RHA-blended cements.

**Figure 6:**
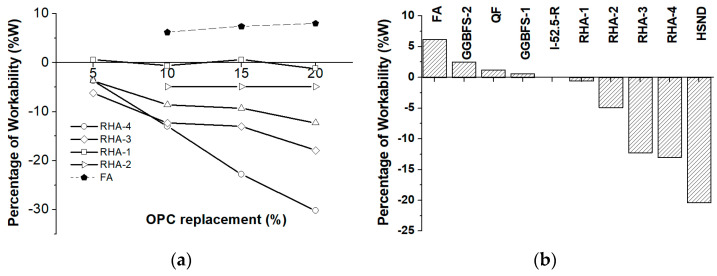
Workability of the mortars using blended cements containing RHA: (**a**) effect of CEM I 52.5 R replacement with RHA and FA; (**b**) effect of the mineral admixture type for 10% cement replacement.

Replacing the cement with different RHA mineral admixtures may modify the mortar’s workability due to the distinct specific surface area, particle morphology and texture, water absorption, reactivity and fineness. Figure 6a presents the workability (%W) percentage related to the control OPC mortar, and %W is calculated as follows:

(2) $$\%W=\frac{W_{i}-W_{c}}{W_{c}}*100$$

where W_i_ is the workability in mm for the mortar prepared with the blended cement; W_c_ is the workability for the control mortar (in this case, using CEM I 52.5 R).

The specific surface area effect was clearly observed when RHA-1 (1.6 m^2^/g) was used rather than RHA-4 (36.5 m^2^/g). The similar specific surface area of RHA-1 to that of cement CEM I 52.5R (1.6 m^2^/g) resulted in the same workability values within the 0–20% replacement range (Figure 6a). RHA-4 (36.5 m^2^/g) had a larger specific surface area than RHA-3 (15.2 m^2^/g), RHA-2 (9.3 m^2^/g) and RHA-1 (1.6 m^2^/g). This parameter significantly influenced the mortar workability values for the cement replacement percentages that equaled or exceeded 5%. These replacement percentages (5–20%) allow the production of non-workable mixtures due to the high absorption/reaction of water [41]. The main reasons for this may be (i) the large specific surface area of the mineral additions, (ii) the pozzolanic reaction where CSH formation on the surface of the RHA grains consumes water; and (iii) the acceleration of cement hydration by nucleation in the grains of the mineral additions. Moreover, the presence of unburned components in the RHA could also influence the workability and the specific surface area [42]. For instance, RHA-2 (9.3 m^2^/g, 4.52% LOI) provided worse consistency than RHA-1 (1.6 m^2^/g, 0.14% LOI).

The workability behavior for the 15–20% blended cements using RHA-4 and RHA-3 means that it is not advisable to prepare this blended cement type with high proportions of these ashes. The upper replacement level could be 10% according to the workability behavior. Thus, some cements, indicated by (*) in Table 3, present problems from a workability point of view.

Figure 4b shows the comparison of the different mineral admixtures in terms of workability for the 10% replacement mortars. The workability of the mortars with 10% RHA-1, RHA-2, RHA-3 and RHA-4 was lower than for those achieved with GGBFS-1 (1.0 m^2^/g) and GGBFS-2 (1.2 m^2^/g), QF (2.6 m^2^/g) and FA (0.8 m^2^/g). The main reasons for these results were the smaller specific surface area of the admixtures other than RHA and, specifically for FA, the spherical morphology of the FA particles. However, the worst workability was observed for NDSF (24.4 m^2^/g) compared to RHA-4 because of its greater fineness (63% in the volume of NDSF particles < 3 µm).

**Figure 7:**
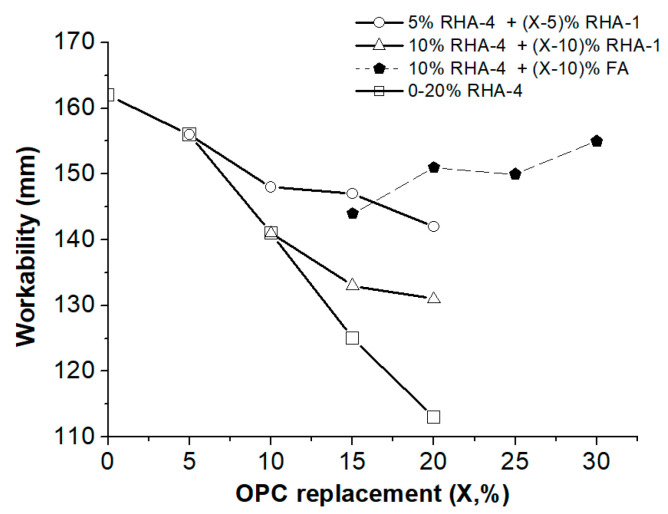
Workability of mortars using ternary blended cements: mixtures RHA4+RHA1 and RHA-4+FA. Comparison to the workability of the mortars containing 5–20% RHA-4.

To prepare blended cements with percentages of cement replacement with RHA higher than 10%, mixtures of the different mineral additions were designed. Mixing RHA-4 and RHA-1 (they presented very different behaviors in workability terms, as previously demonstrated) allowed a higher replacement percentage to be applied, and the workability improved compared to the sole use of reactive RHA-4. Figure 7 shows the workability of the mortars with different proportions of RHA-4 and RHA-1. Low workability (113 mm) was obtained using the 20% RHA-4 replacement. To enhance the workability, the two mixtures of 5% RHA-4 plus 5–15% RHA-1 and 10%RHA-4 plus 5–10% RHA-1 were tested. The workability for the 20% replacement mortars increased, and the following workability values were obtained: 142 mm (for 5%_RHA-4_ + 15%_RHA-1_) and 131 mm (for 10%_RHA-4_ + 10%_RHA-1_). These values were significantly higher than those for 20%_RHA-4_. For the tested mixtures, the workability of the mortars obtained by mixing RHA-4 and RHA-1 showed a linear decrease with the amount of replaced cement, and no synergic effect took place. This workability study demonstrates that preparing mixtures containing both a large and small specific surface area could be promising for the valorization of different RHA types.

The mixture of different types of pozzolans can produce synergic effects as a result of the individual properties of each one [43]. This fact was noted when highly reactive RHA-4 and FA were used together. For instance, the worsening of the workability when employing RHA-4 with a large specific surface area could be notably improved by FA blending. Figure 6a shows (dashed line) the workability of the mortars with a 10–20% level of cement replacement with FA: a slight increase in %W occurred with the rising replacement percentage. Figure 7 depicts (dashed line) the workability improvement upon increasing the quantity of FA added to RHA-4. The 20% replacement system with 10%_RHA-4_ + 10%_FA_ yielded 151 mm workability and the 30% replacement one with 10%_RHA-4_ + 20%_FA_ yielded 155 mm. These values are equivalent to those obtained for the 5%_RHA-4_ system. The main reasons for the improved workability with FA addition are the smaller specific surface area and the high sphericity of the FA particles compared to CEM I 52.5 R. The obtained results demonstrate an interesting strategy to compensate for the reduction in workability by using RHA with a large specific surface area and adding a mineral admixture with a ball-bearing effect [44].

**Figure 8:**
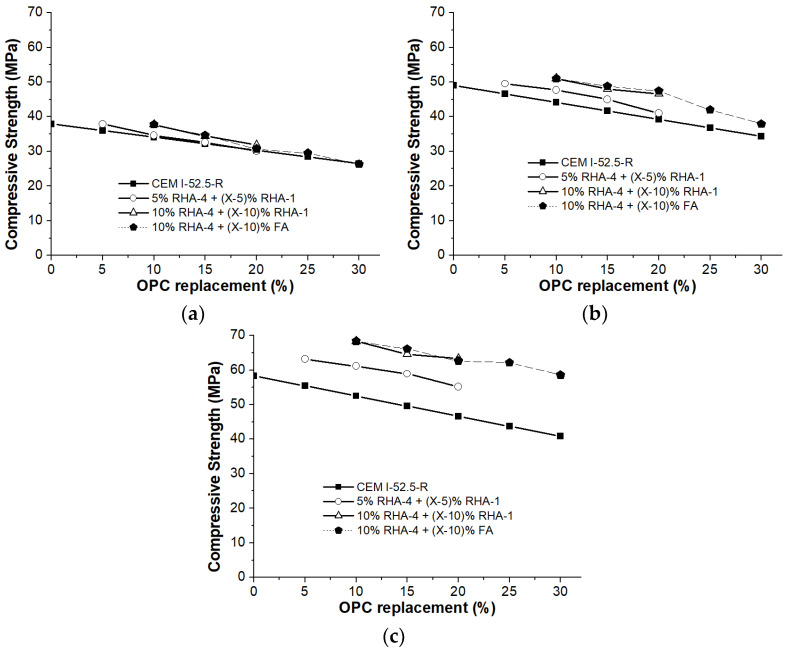
Compressive strength development for the mortars containing ternary blended cements with RHA-4 for the curing times of (**a**) 2 days, (**b**) 7 days and (**c**) 28 days. The values for the CEM I 52.5 R mortars were calculated by multiplying the control mortar by the cement content, i.e., (100 − %OPC) replacement.

From previous workability studies, it can be concluded that employing certain mixtures of mineral additions can be appropriate for the preparation of mortars with good workability. However, compressive strength tests must be carried out. Some mixed mineral admixtures, i.e., RHA-4+RHA-1 and RHA-4+FA, were also studied from a mechanical point of view. The compressive strength values for the mortars cured at 2, 7 and 28 days were measured. Figure 8 offers the compressive strength values for the mortars with 5–20% replacement levels. Two sets of mixtures of RHA-4+RHA-1 were studied: one containing 5% RHA-4 and the other one containing 10% RHA-4. The 10%_RHA-4_ + (X − 10%)_FA_ mixtures (where X = 20, 25 and 30%) were also tested. In Figure 8, the lines corresponding to the control mortar (CEM I 52.5 R) represent the theoretical linear decrease according to the cement content in the cement-replaced mortars.

For the 2- and 7-day curing times, most samples obtained similar or lower compressive strength values than those found for the control mortar. However, all of the cement-replaced samples showed equal or higher strength values compared to the corresponding one when taking into account the amount of CEM I 52.5 R in these mixtures. This fact suggests that blending admixtures significantly contributes to the strength at these early ages.

Except for mixture 5%_RHA-4_ + 15%_RHA-1_, the compressive strength values at 28 curing days were similar to or higher than those for the 100% OPC control specimen. This means that the strength development contribution of the mixture of pozzolanic additions was very important at the 28-day curing time. Thus, it is possible to prepare ternary blended cements with 30% less OPC using the selected mixtures of types RHA-4+RHA-1 and RHA-4+FA.

The compressive strength values for the ternary blended cements with either siliceous FA or RHA-1 were similar at 28 curing days. However, when using FA, the consistency was better (151 mm, 10%_RHA-4_ + 10%_FA_) compared to when RHA-1 was employed (131 mm, 10%_RHA-4_ + 10%_RHA-1_). This fact suggests the positive effect of FA addition on RHA-containing ternary cements.

According to these results, some standard ternary blended cements could be prepared with high-reactivity RHA and with either siliceous FA or low-reactivity RHA; these are listed in Table 4. Some cements are proposed according to the compressive strength values, as well as a minimum workability value for the mortar (the proposal had 140 mm, which corresponded to reduced workability by ca. 15% vs. the CEM I 52.5 R mortar). All of the ternary cements that can be produced belong to the 52.5 strength category. Thus, the CEM II/A-H 52.5 cements can be prepared by blending 5%/5%, 5%/10% or 5%/15% of high-/low-reactivity RHA. This demonstrates that low-reactivity RHA valorization is feasible. Moreover, cements of type CEM II/A-(H-V) can be designed with high-reactivity RHA with 10% content. Additionally, some blended cements with replacement percentages above 20% can be prepared by blending RHA and FA, and, consequently, the CEM-II B (H-V) ternary cements can be prepared.

## 4. Conclusions

Replacing cement with a mineral admixture modifies the workability of the blended cement: it decreases as more OPC (CEM I 52.5 R) is replaced with RHA-4 with a large specific surface area and high amorphous silica content.

RHA-4 is feasible for the production of standard blended cements of the CEM II/A type if the optimum quantity of 10% replacement is applied due to the workability limitations. CEM II/A-H would be produced (where “H” indicates RHA as a mineral component) with the same strength performance as CEM I.

RHA-4 mixtures with other inorganic additions (FA or low-reactivity RHA) are adequate for the preparation of ternary blended cements, such as CEM II/A and CEM II/B, with improved compressive strength at early (2 and 7 days) and 28-day curing ages. The significant contribution to the compressive mechanical strength due to the pozzolanic activity of high-reactivity RHA-4 favors mixtures with materials with lower pozzolanic reactivity, such as crystalline RHA (RHA-1) or FA. Cement replacement values of up to 30% are proposed and some standardized cements of types CEM II/A (H-V) and CEM II/B (H-V) can be designed.

In summary, in view of these results, which serve as an example, the Portland cement industry has the potential to implement cements with new mineral additions that valorize waste (for example, waste from agricultural activities), in compliance with the required standards of quality.

## Figures and Tables

**Table 1:** Main physico-chemical properties of CEM I 52.5 R (OPC), the RHA samples and the other mineral admixtures tested in this research.

Material	Amorphous Silica (%) ^#^	Specific Surface Area (m^2^/g) ^&^	Loss on Ignition LOI (%)	Particle Size Distribution (%, by Weight)		
				<3 µm	3–32 µm	>32 µm
CEM I 52.5 R	Nd *	1.6	0.98	10.9	83.8	5.3
RHA-1	11.7	1.6	0.14	29.8	62.4	7.8
RHA-2	12.0	9.3	4.52	23.2	62.6	14.2
RHA-3	71.0	15.2	8.24	13.3	71.5	15.2
RHA-4	100.0	36.5	17.65	13.0	72.8	14.2
FA	Nd *	0.8	0.66	34.0	53.7	12.3
NDSF	100	24.4	2.94	63.0	34.3	2.7
GGBFS-1	Nd *	1.0	<0.1	10.8	75.9	13.3
GGBFS-2	Nd *	1.2	<0.1	14.8	71.1	14.1
QF	Nd *	2.6	<0.1	4.9	83.9	11.2

^#^ Amorphous silica obtained by the method described in [27]; amorphous silica in relation to the total silica. * Nd: not determined; ^&^: obtained by BET.

**Table 3:** Designation of the standard blended cements with different RHA reactivity.

RHA	Replacement Percentage (%, by Weight)			
	5%	10%	15%	20%
RHA-4	CEM II/A-H 52.5 R	CEM II/A-H 52.5 R	CEM II/A-H 52.5 R ^(^*^)^	CEM II/A-H 52.5 R ^(^*^)^
RHA-3	CEM II/A-H 52.5 R	CEM II/A-H 52.5 R	CEM II/A-H 52.5 N	CEM II/A-H 52.5 N ^(^*^)^
RHA-1	CEM II/A-H 52.5 R	CEM II/A-H 52.5 N	CEM II/A-H 42.5 R	CEM II/A-H 42.5 R

^(^*^)^ Mortars with considerably reduced workability (>15%) in relation to the control mortar (see Section 3.5 for more details).

**Table 4:** Designation of cements containing high-reactivity RHA and low-reactivity RHA or FA.

Nomenclature	Ternary Blended Cement
CEM II/A-H 52.5 R	5% RHA-4 + 5% RHA-1
	5% RHA-4 + 10% RHA-1
CEM II/A-H 52.5 N	5% RHA-4 + 15% RHA-1
CEM II/A-(H-V) 52.5 R	10% RHA-4 + 5% FA
CEM II/A-(H-V) 52.5 N	10% RHA-4 + 10% FA
CEM II/B-(H-V) 52.5 N	10% RHA-4 + 15% FA
	10% RHA-4 + 20% FA

**Figure A1:**
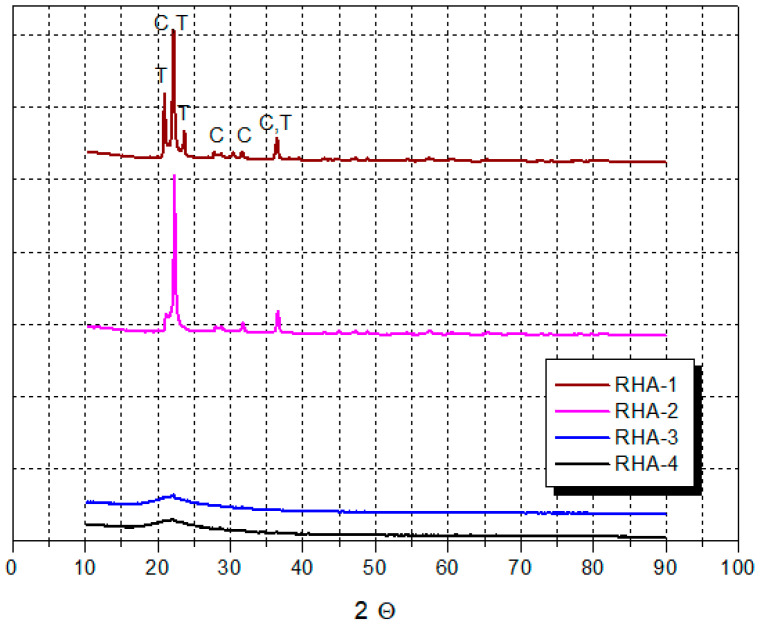
X-ray diffraction patterns of RHA samples (Key: C: Cristobalite; T: Tridymite).

## Data Availability

The original contributions presented in the study are included in the article, further inquiries can be directed to the corresponding author.

## Appendix A

**Table A1:** XRF data for RHA samples (weight %).

	RHA-1		RHA-2		RHA-3		RHA-4	
	%	Std	%	Std	%	Std	%	Std
SiO_2_	94.35	0.65	89.12	0.45	86.12	0.04	78.00	0.10
Al_2_O_3_	0.52	0.00	0.27	0.00	0.20	0.00	0.16	0.00
Fe_2_O_3_	0.08	0.00	0.09	0.00	0.39	0.00	0.31	0.01
CaO	1.02	0.00	1.20	0.01	1.26	0.00	0.98	0.00
MgO	0.47	0.00	0.54	0.00	0.41	0.00	0.45	0.00
SO_3_	0.10	0.02	0.19	0.06	0.21	0.01	0.12	0.02
Na_2_O	0.14	0.01	0.35	0.01	0.18	0.02	0.24	0.02
K_2_O	2.14	0.05	2.95	0.06	2.52	0.02	1.92	0.07
TiO_2_	0.09	0.00	0.09	0.00	0.08	0.00	0.07	0.00
Mn_2_O_3_	0.05	0.00	0.06	0.00	0.09	0.00	0.08	0.00
LOI	0.42	0.02	4.69	0.02	8.51	0.05	17.65	0.02

## Appendix B

XRD diffractograms for RHA samples.

## Appendix C

SEM micrographs for non-ground RHA samples.
